# Methamphetamine Use Among Iranian Heroin Kerack-Dependent Women: Implications for Treatment

**DOI:** 10.5812/ijhrba.10216

**Published:** 2013-06-26

**Authors:** Zahra Alam Mehrjerdi, Zohreh Abarashi, Sahar Mansoori, Abbas Deylamizadeh, Javad Salehi Fadardi, Alireza Noroozi, Mehran Zarghami

**Affiliations:** 1 Iranian National Center for Addiction Studies (INCAS), Tehran University of Medical Sciences, Tehran, IR Iran; 2 Center of Psychological Counseling Services, Ferdowsi University, Mashhad, IR Iran; 3 Division of Research on Drug Use and Dependence, Rebirth Society (NGO), Tehran, IR Iran; 4 Department of Psychology, Faculty of Psychology and Educational Sciences, Ferdowsi University, Mashhad, IR Iran; 5 School of Advanced Technologies in Medicine (SATM), Iranian National Center for Addiction Studies (INCAS), Tehran University of Medical Sciences, Tehran, IR Iran; 6 Department of Psychiatry, Faculty of Medicine, Mazandaran University of Medical Sciences, Sari, IR Iran; 7 Psychiatry and Behavioral Sciences Research Center, Mazandaran University of Medical Sciences, Sari, IR Iran

**Keywords:** Methamphetamine, Heroin kerack, Women, Treatment

## Abstract

**Background:**

Co-use of heroin kerack with methamphetamine (MA) is a new epidemic health concern among Iranian female drug users. Yet, little is known about this issue because of stigma.

**Objectives:**

The current study aimed to investigate the reasons associated with initial and continued co-use of heroin kerack with MA among two groups of regular and recreational female co-users, their motivations associated with treatment entry and to compare their general characteristics at a drop in center (DIC) in Tehran.

**Materials and Methods:**

82 clients were randomly recruited. A researcher-designed questionnaire was used to collect data. Data was analyzed by performing descriptive statistics, the Chi-square test and t-test.

**Results:**

The mean age of the participants were 31 (SD = 8) years. Reducing negative affect (55%), addicted family and social networks (50%), curiosity (48%), and the lack of knowledge on addictive effects of co-use of heroin kerack with MA (32%) were the most frequently reported reasons at initiation. Drug dependence (71%) and drug availability (56%) were reasons of continued co-use. Restoring health (61%), fear from becoming MA abuser only (33%), and fear from making a transition from heroin kerack and MA smoking to injection (15%) were important motivations for treatment entry. Regular co-users were more likely to be single (41.7% vs. 14.7%, P < 0.001), jobless (45.8% vs. 38.2, P < 0.05), homemaker (50% vs. 35.3%, P < 0.01), recently incarcerated (16.7% vs. 11.7%, P < 0.01), and were less likely to be enrolled in opioid replacement programs (33.5% vs. 41%, P < 0.01). Regular co-users were younger (30.6 vs. 32.1 years, P < 0.05), less educated (9.6 vs. 10.8 years, P < 0.05) and had a longer duration of drug dependence (9.6 vs. 8 years, P < 0.05).

**Conclusions:**

Reasons associated with initial and continued co-use of heroin kerack with MA, factors associated with treatment entry and the differences between regular and recreational co-users should be specifically considered in designing and tailoring drug use treatment programs for this group.

## 1. Background

Iran borders Afghanistan, the main opium producer in the Middle East region (1). Studies show that Iran has the highest per capita opioid use in the world (1). In 2008, Iran led all countries with 23% of all heroin seizures in the world due to its important geographical situation on the main drug trafficking routes (2). In recent years, a new locally produced synthetic heroin which is colloquially named heroin kerack has hit Iranian illicit drug market and its abuse is epidemic among Iranian drug users (3). Heroin kerack (not to be mistaken with crack cocaine) belongs to Iran only and contains diacetylmorphine mixed with some adulterants including acetylcodeine, 6-monoacetylmorphine, caffeine, papaverine, noscapine, dextromethorphan, morphine, codeine, phenobarbital and diazepam (3). The main route of heroin kerack abuse is smoking but, the appearance of heroin kerack as a purified and highly potent form of heroin has been also cited as a potent driver of injection practice in Iran (4).There is evidence that heroin kerack and MA abuse have become critical health concerns among drug abusers in Iran. A study at the methadone maintenance treatment clinic of Baharan psychiatric hospital in Zahedan city, in the east of Iran showed that MA abuse increased from 6% among the patients in 2009 to approximately 20% in 2011 (5). Another study on 810 drug abusers referring to methadone clinics in different parts of Tehran showed that 24% were heroin kerack-dependent clients (6). Some drugs such as MA are frequently co-abused with other drugs, including alcohol (7), marijuana and depressants (8). The combinations of MA with other drugs containing heroin are assumed to reach a “relaxed high” or to negate some of negative impacts of high-dose amphetamine use such as agitation and irritability (9). A study showed that reducing negative affect is a strong motivation to co-abuse heroin with MA (9). An increasing number of studies have suggested that as a psychostimulant drug, MA abuse poses a serious health risk on illicit opioid users (10-12), especially on women but Iranian women experience greater social stigma from being drug abusers and are less likely to seek treatment (13). As a result, no considerable information on female drug abusers is available in Iran (4). A recent study on 97 Iranian women at a female-specific methadone clinic in Tehran showed that 24% were heroin abusers and 4% were MA abusers and many clients reported making a transition from traditional patterns of opium abuse to the new pattern of heroin abuse (e.g. heroin kerack) (14). In 2007, a study on 78 women at a free methadone clinic with ancillary services for female drug abusers in the south of Tehran showed that opium and heroin abuse (e.g. heroin kerack) was reported by 69% (15). Co-use of heroin kerack with MA could have critical health implications but there is a paucity of research on this issue in Iran especially on drug-abusing women who recreationally or regularly co-use heroin kerack with MA. As a result, motivations and reasons associated with initial and continued co-use of heroin kerack with MA are unknown and the treatment needs of this vulnerable group are unrecognized.

## 2. Objectives

The current study aims to investigate the reasons associated with initial and continued co-use of heroin kerack with MA, motivations associated with treatment entry and to compare the general characteristics of two groups of regular and recreational female co-users at a DIC in Tehran.

## 3. Materials and Methods

### 3.1. Design and Setting

Between March and September 2009, a cross-sectional study was conducted at a female-specific DIC in the south of Tehran (a low socio-economic area with problematic drug use problem. Of 119 drug users referring to the DIC, 82 participants were co-users of heroin kerack with MA and were randomly recruited. After providing informed consent, participants were interviewed by forth well-trained female psychologists.

### 3.2. Participants

Participants were heroin kerack-dependent clients who were regular MA users (n = 48) or recreational MA users (n = 34) within the 12 months before treatment entry. Eligibility criteria included 1) ≥ 18 years old, 2) reported regular or recreational co-use of heroin kerack with MA within the past 12 months before treatment entry, 3) living in Tehran at the time of study, and 4) signing consent form to participate in the study. Exclusion criteria included 1) withdrawal and or intoxication symptoms, and 2) disagreement with signing consent form.

### 3.3. Measures

A questionnaire was designed to conduct interviews. The items of the questionnaire were elicited from the Addiction Severity Index (5th Edition) (16), the questionnaire of the “WHO Drug Injection Study Phase II (Version 2b) (17), and according to the knowledge of our interviewers on co-use of heroin kerack with MA among Iranian women. Three psychiatrists also adapted the questionnaire according to the situation in Iran, which was followed by a pretest assessment on a sample of women treatment seekers in the south of Tehran. A two-week pre-test, post-test assessment of the questionnaire on 15 women showed high reliability (r = 93%) to meet the study aims. The validity of the questionnaire was obtained from reviewing the previous studies (16, 17). The questionnaire included assessments of socio-demographic characteristics, substance use and injecting drug use practices, high risk behaviors, factors associated with initial and continued co-use of heroin kerack with MA, and motivations for treatment entry.

### 3.4. Analysis

Statistical analyses were performed using SPSS for Windows (version 16.0, 2007; SPSS Inc., Chicago, IL, USA). Descriptive statistics characterized demographic characteristics, substance use details, high risk behaviors, initial and continued co-use of heroin kerack with MA, and motivations for treatment entry. We compared the differences between the two groups using the Chi-square test for categorical variables and t-test for continuous variables.

### 3.5. Ethical Considerations

The protocol of the study was approved by the Institutional Review Board of Tehran University of Medical Sciences in Tehran, Iran. Interviews were conducted individually and participants were assured of the confidentiality of interviews. An identification code was included on each questionnaire. Participants were ensured that non-participation in the study would not affect their treatment and harm-reduction service utilization.

## 4. Results

### 4.1. Demographic Characteristics

82 participants who had enrolled for treatment participated in the study. The mean age of the participants were 31 (SD = 8) years (range: 18 - 47 years). 81.7% had < 12 years of education. Mean years of education was 10 (SD = 8) years. 40.2% were married. 42.7% were jobless. Participants reported having children between none and four children (Table 1).

**Table 1.** Demographic Characteristics of the Participants (n = 82)

Variable	Regular co-users, N=48	Recreational co-users, N=34	Total, N =82
**Age range, year**	18-36	18-47	18-47
**Age, Mean ± SD**	30.6 ± 5.1	32.1 ± 11.2	31 ± 8
**Education, No. (%)**			
<12 years	43 (89.6)	24 (70.5)	67 (81.7)
12 years	4 (8.3)	8 (23.5)	12 (14.7)
14-16 years	1 (2.1)	2 (6)	3 (3.6)
**Education, Mean ± SD**	9.6 ± 6.5	10.8 ± 9.8	10 ± 8
**Marital status, No. (%)**			
Married	16 (33.3)	17 (50)	33 (40.2)
Single	20 (41.7)	5 (14.7)	25 (30.5)
Separated/divorced	12 (25)	12 (35.3)	24 (29.3)
**Living conditions, No. (%)**			
With family	45 (93.7)	30 (88.2)	75 (91.4)
No stable living condition	3 (6.3)	4 (11.8)	7 (8.6)
**Job status, No. (%)**			
Homemaker	24 (50)	12 (35.3)	36 (43.9)
Jobless	22 (45.8)	13 (38.2)	35 (42.7)
Employed	2 (4.2)	9 (26.5)	11 (13.4)
**Number of Children**	0-2	0-4	0-4

### 4.2. Substance Use and High Risk Behaviors

The Onset age of drug use was 17 years (SD = 9). Mean duration of drug dependence was nine years (SD = 9). Multiple drug detecting urine tests which had been registered by the physician of the DIC at registration in their clinic files confirmed their self-report of co-use of heroin kerack with MA. Regular users reported that they co-used heroin kerack with MA in 25.3 days (SD = 3, range: 20 - 30 days) in the past 12 months before treatment entry. Recreational users reported that they co-used heroin kerack with MA in eight days (SD = 3, range: 5 - 11 days) in the past 12 month before treatment entry. Duration of participation in the current treatment was 9.3 (SD = 11) months. 32% reported lifetime sex work. 28% reported sex with others for drug and/or money in the past 12 months. 43% reported lack of lifetime condom use. 76% were sexually active in the past months while only 31% reported condom use in the past month (See details in Table 2).

**Table 2.** Substance Use and High Risk Behaviors Among Participants (n = 82)

Variable	Regular co-users, N=48	Recreational co-users N=34	Total, N=82
**Onset age of drug use, y, Mean ± SD**	17 ± 7	16.6 ± 12	17 ± 9
**Age range, y**	13-32	16-39	13-39
**Duration of drug dependence, y, Mean ± SD**	9.9 ± 10	8 ± 8	9 ± 9
**Initial substance use, No. (%)**			
Alcohol drinking	10 (21)	11 (32.3)	21 (26)
Hashish smoking	10 (21)	9 (26.5)	19 (23)
Opioids	27 (56)	12 (35.2)	39 (47)
**Other substances**	1 (2)	2 (6)	3 (4)
**Duration of drug dependence, y, Mean ± SD**	9.8 ± 8	8.1 ± 11	9 ± 9
**Main route of co-use of Kerack with MA, No. (%)**			
Smoking	47 (98)	32 (94)	79 (96)
Injection	1 (2)	2 (6)	3 (4)
**Presence in the current treatment, month, Mean ± SD**	9.5 ± 10	9.1 ± 12	9.3 ± 11
**Number of lifetime drug use treatment, Mean ± SD**	5 ± 7.1	6.3 ± 8.3	5.6 ± 8
**Recent incarceration, Past 2 y, No. (%)**	8 (16.7)	4 (11.5)	12 (14.6)
**Recently enrolled in opioid replacement program**	16 (33.3)	14 (42)	30 (36.6)
**Lifetime sex work**	13 (27)	13 (38)	26 (32)
**Sex for drug or money, Past 12 months**	12 (25)	11 (32.3)	23 (28)
**Condom use, Past 12 months**	19 (39.5)	16 (47)	35(43)
**Number of sexually active cases, past months**	30 (62.5)	32 (94.1)	62 (76)
**Condom use among sexually active cases**	10 (33.3)	9 (28.1)	19 (31)

### 4.3. Initial and Continued Co-use of Heroin Kerack With MA and Treatment Entry

Participants reported different motivations associated with initial co-use of heroin kerack with MA. 55% reported that they co-used heroin kerack with MA to reduce negative affect. 50% reported that their addicted families and social networks were important factors at initiation. 48% reported that curiosity was an important initial factor. 32% reported that they lacked knowledge on addictive effects of co-use and assumed that MA was a non-addictive drug which could be used for quitting heroin kerack. As a result, by co-use, they attempted to regularly substitute MA use for stopping heroin kerack use. Participants reported two main reasons for continued co-use included drug dependence (71%), and drug availability (56%). Participants were motivated to enter treatment for restoring health (61%) followed by fear from being MA abuser only (33 %), and fear from making a transition from heroin Kerack and MA smoking to injection (15%).

### 4.4. Regular Co-users vs. Recreational Co-users

Regular co-users were more likely to be single (41.7% vs. 14.7%, P < 0.001), jobless (45.8% vs. 38.2, P < 0.05), homemaker (50% vs. 35.3%, P < 0.01), recently incarcerated (16.7% vs. 11.7%, P < 0.01), and were less likely to be enrolled in opioid replacement programs (33.5% vs. 41%, P < 0.01). Regular co-users were younger (30.6 vs. 32.1 years, P < 0.05), less educated (9.6 vs. 10.8 years, P < 0.05) and had a longer duration of drug dependence (9.6 vs. 8 years, P < 0.05) (Table 3). No relationship was found between the other study variables.

**Table 3.** Differences Between Regular and Recreational Co-users (n = 82)

Variables	Regular co-usersn = 48No, (Percent/Mean)	Recreational co-usersn = 34No. (Percent/Mean)	P - value
**Marital status (single)** ^a^	20(41.7)	5 (14.7)	< 0.001
**Job status (jobless)** ^a^	22 (45.8)	13 (38.2)	< 0.05
**Job status (home maker)** ^a^	24 (50)	12 (35.3)	< 0.01
**Recently incarcerated** ^a^	8 (16.7)	4 (11.7)	< 0.01
**Recently enrolled in opioid replacement programs** ^a^	16 (33.5)	14 (41)	< 0.01
**Age (Mean Years)** ^a^	48 (30.6)	34 (32.1)	< 0.05
**Education (Mean Years)** ^a^	48 (9.6)	34 (10.8)	< 0.05
**Duration of drug dependence (Mean Years)** ^a^	48 (9.6)	34 (8 )	< 0.05

^a^Only statistically significant variables (P < 0.05) have been reported in Table 3.

## 5. Discussion

As a distinct aspect of Iran`s drug culture, co-use of heroin kerack with MA is a newly emerged health problem in Iran especially among women but literature is not well-documented on etiology and treatment. The study findings showed that participants were commonly young, married, homemaker, and with low level of education. Findings of the current study are in contrast with an American study showed that MA users` median education level was graduation from high school. Marital status of most participants was single or separated and women were much less likely to report full time employment and unemployment (18). Findings of the current study may be indicative of the traditional roles of women in a developing country. Additionally, the current study participants initiated drug use at a young age with drugs of less detrimental effects such as hashish and then made a transition to co-use of heroin kerack with MA as drugs of more detrimental impacts on health. Findings of the current study showed that the participants experienced a transition from the traditional patterns of drug use in Iran to new patterns of drug use at relatively a young age and rapidly experienced continued use and dependence. A study showed that an earlier initiation of drug use is associated with more possibility for continued drug use (19). The transition from traditional drugs of abuse to new ones and co-use of heroin kerack with MA among these women should be considered for treatment because there is evidence that the current trend is increasing to some extent as rapidly as that of men. Studies in other countries showed that high use rates of some drugs are emerging for women, equivalent to those of men (20). High risk behaviors such as sex work, unprotected sex, and drug injection were reported by a group of the current study participants which are subjects to training in safe sex, safe injection and prevention. A study showed that MA use increased the risk for engaging in multiple sex partners, and risky sexual intercourse among adults 18 - 24 years old (21). High risk behaviors among participants are likely to be partly due to MA use and deserve further research. Participants reported that heroin kerack use had negative depressant impacts on their everyday life. Apparently, there is no study to show that the depressant effects of heroin kerack use would be inevitably negated by using MA, or vice versa. Combining drugs that individually have a complex effect on physical and psychological aspects inevitably complicates things even further, making these executive operations less predictable and more subject to error among users. Findings of the current study showed implications for immediate drug education. Curiosity was an important initial factor for co-use of heroin kerack with MA too. Witteveen et al. (2007), studied factors associated with initiation of cocaine and heroin in problem drug users in Amsterdam, the Netherlands, and found that curiosity was an important facilitating factor (22). Another study on initiation of MA use among 48 young Thai MA users in Chiang Mai city and the suburbs emphasized the role of curiosity at initiation (23). Lack of knowledge on addictive effects of MA use and assuming that MA was a non-addictive drug which could be used to quit heroin kerack was another important reason at initiation. This issue was followed by MA substitution to quit heroin kerack use and is a new study finding in the current study. A study on 352 MA users in the US showed that later MA order in the initiation sequence was partly related to initiating MA to substitute for another drug (24). Witteveen et al. (2007) emphasized the role of misinformation in their study on initiation of cocaine and heroin use in drug users in the Netherlands (22). Dependence on co-use of heroin kerack with MA and drug availability was two reasons for continued co-use of heroin kerack with MA. The Study on the etiology of continued drug use among women is not well-documented. Some sporadic studies showed that women used heroin more frequently or more likely to be diagnosed with heroin dependence compared with men (25), and drug availability is an important reason for drug use among women (26). This issue recognizes urgent prevention and treatment programs at different levels of these women`s lives especially within individual and community contexts. Detrimental physical and psychological effects of co-use of heroin kerack with MA were important motivations for some participants to enter treatment. This is consistent with an old study in Sweden showed that women were more likely to seek treatment after serious acute complications of their drug use such as unconsciousness (27). This study finding implicates implementing prevention and treatment programs for these women. The current study participants frequently reported that although they combined MA use with their regular heroin kerack use but they believed that they were at risk for making a transition from co-use of heroin kerack with MA to MA abuse only. No study was found to compare the current study findings with, but this issue implicates urgent drug use prevention programs for these women. Fear from making a transition from smoking heroin kerack with MA to injection was another important factor for treatment entry which should be considered in designing treatment and harm reduction programs for this group of drug users. This issue may be partly due to high stigma that the Iranian community imposes on injection and social observations that these women had from cases that made a transition from drug smoking to drug injection. No study was also found to compare this finding with, but this issue implicates implementing prevention programs that target those aspects of drug use which these women consider important. The descriptions of regular and recreational co-users showed that some demographic and drug use-related characteristics were different between regular and recreational users which are likely to make them vulnerable to the regular pattern of co-using heroin kerack with MA. Findings of the current study can provide a basis to develop prevention and treatment strategies based on the patterns of co-use of heroin kerack with MA and should be specifically considered in designing and tailoring treatment programs for recreational and regular co-users of heroin kerack with MA. The current study confirms the importance of certain demographic and drug use characteristics associated with co-use of heroin kerack with MA. These issues were likely to lead some of heroin kerack-dependent participants to a high risk situation to co-use heroin kerack with MA regularly but further research is required to assess the nature of these associations. As the MA epidemic among Iranian female heroin Kerack users continues to grow, the need for research on etiology and effective treatment outcomes increase. Moreover, drug-using women experience stigma of being drug users and are reluctant to seek treatment (13). therefore, this could be particularly true concerning female-specific treatment. Some studies conducted in recent years, have shown that drug use treatment programs specifically developed for women have increased effective treatment outcomes (28). Implementing comprehensive treatment programs addressing different personal, familial, social and cultural needs of women have become an important practice (28). These results should be interpreted within the limitations of the study. The study findings emphasize some important reasons associated with initiation, continued use of heroin kerack with MA, motivations to enter treatment and some differences between regular and recreational co-users of heroin kerack with MA that may have implications for female-specific research as well as for prevention and treatment. Because of the exploratory nature of the current study, the results of this study cannot be generalized to other female heroin kerack and MA co-users in Iran. Further studies with more representative samples are suggested.
